# Genotoxicity assessment of mesoporous silica and graphene oxide in GDL1 cells

**DOI:** 10.1186/s41021-025-00350-y

**Published:** 2025-12-23

**Authors:** Rikako Ishigamori, Akiko Ohno, Kiyoshi Fukuhara, Shinya Hasegawa, Yukari Totsuka

**Affiliations:** 1 Environmental Health Sciences, Hoshi University, 2-4-41 Ebara, Shinagawa-Ku, Tokyo, 142-8501 Japan; 2 Division of Genome Safety Science, National Institute of Health Sciences, 3-25-26 Tonomachi, Kawasaki-ku, Kawasaki City, Kanagawa 210-9501 Japan; 3 School of Pharmacy, Showa Medical University, 1-5-8 Hatanodai, Shinagawa-ku, Tokyo, 142-8555 Japan

**Keywords:** Mesoporous silica, Graphene oxide, Genotoxicity

## Abstract

**Background:**

Nanomaterials such as mesoporous silica and graphene oxide are increasingly used in industrial, medical, and cosmetic applications due to their unique physical and chemical properties. However, their potential genotoxicity remains poorly understood. To evaluate the associated health risks of mesoporous silica and graphene oxide, we assessed their cytotoxicity and genotoxicity in GDL1 cells using trypan blue exclusion and *gpt* mutation assays, followed by mutation frequency and spectrum analysis through *gpt* gene sequencing.

**Results:**

A 24-hour exposure of mesoporous silica to GDL1 cells induced dose-dependent reductions in cell viability, as well as dose-dependent increases in *gpt* mutation frequencies at 0.06 and 0.09 mg/mL. Graphene oxide induced cytotoxicity at higher concentrations (0.2 and 0.4 mg/mL) and significantly increased *gpt* mutation frequency in the highest concentration exposure group compared to controls. Mutation spectrum analysis revealed a significant increase in G: C to A: T transitions in both the exposed groups. In addition, exposure to mesoporous silica significantly increased G: C to T: A transversions, while graphene oxide exposure significantly increased G: C to C: G transversions. Mutation hotspots at positions 64, 164, and 416 in the *gpt* gene were identified exclusively in the mesoporous silica-treated group, indicating material-specific mutagenesis. Mutations at position 401 were detected exclusively in the graphene oxide group, indicating this site as a potential mutation hotspot.

**Conclusion:**

These results demonstrate that both mesoporous silica and graphene oxide exhibit cytotoxic and genotoxic potential in vitro. The mutation patterns suggest that oxidative DNA damage, as well as inflammation associated with oxidative stress, may contribute to the observed mutagenicity. The findings reported here provide valuable insights into the molecular mechanisms underlying the mutagenicity induced by these nanomaterials and contribute to the assessment of potential human health risks.

## Introduction

Nanomaterials are defined as materials with at least one dimension in the size range from approximately between 1 and 100 nm and have unique physical, biological, and chemical characteristics [1]. A wide variety of nanomaterials have already been used for several decades and are applied in various applications for industrial, medical, and cosmetic fields because of their useful properties. Among the various nanomaterials, mesoporous silica has attracted a great deal attention due to its exceptional properties such as uniform pore size, very large surface area, ease of surface modification, high biocompatibility and biodegradability, high mechanical and thermal stability and stable aqueous dispersibility [2, 3]. These advantageous properties make mesoporous silica highly suitable for applications in catalysis, drug delivery systems (DDS), and diagnostic imaging [4, 5]. Graphene oxide has also garnered considerable interest in biological and medical research due to its unique physical and chemical properties [6]. Graphene oxide is a material that can bond with oxygen-containing functional groups (epoxy, hydroxy, carboxyl, and carbonyl groups) on its surface and has attracted considerable attention [7]. These functional groups facilitate surface modifications, allowing for the attachment of various biomolecules (e.g., proteins, DNA, RNA) and chemical compounds. Consequently, graphene oxide has been utilized as a biosensor surface and in DDS platforms [8–11]. Other biomedical applications include photothermal therapy [12] and tissue engineering [13]. Given the increasing application of these substances for human use, their potential release into the environment, and subsequent human exposure, the evaluation of their human health effects has become a key focus in recent years. However, with limited knowledge regarding their safety, there is an urgent need for a human health risk assessment of these materials.

Previously, we have examined the genotoxicity of some nanomaterials by using both in vitro and in vivo assays and reported genotoxic and other toxic properties of them [14–17]. In the field of genetics, genotoxicity is an important word and refers to the property of chemical agents that are able to alter genetic information. In this study, we focused on mutagenicity as an endpoint of genotoxicity and examined mutations using the gpt assay. When a mutation is present in a germ cell, it can be inherited and potentially lead to genetic disorders in the next generation. In somatic cells, mutations in critical genes may lead to cancer development. Thus, every mutagen is considered potentially carcinogenic. Despite the increasing application of mesoporous silica and graphene oxide, their genotoxic potential remains poorly understood. Therefore, it is necessary to determine whether these nanomaterials can induce mutations, and to elucidate the mechanisms underlying their genotoxicity.

One of the major genotoxic events induced by chemicals are point mutations. To assess such mutations in vitro, the *gpt* delta L1 (GDL1) cell line was established by Takeiri et al. from lung fibroblasts of *gpt* delta transgenic mice through transfection with the SV40 T antigen [18]. This cell line is a valuable tool for the detection of point mutations, similar to *gpt* delta transgenic mouse models [18]. In the present study, we evaluated the cytotoxicity and genotoxicity of mesoporous silica and graphene oxide using the GDL1 cell line, aiming to obtain fundamental data for elucidating their potential health risks.

## Materials and methods

### Materials

Mesoporous silica (Silica, mesostructured, MCM-41; Sigma-Aldrich, St. Louis, MO, USA) was kindly provided by the National Institute of Health Sciences (Kanagawa, Japan). It consists of silicon dioxide and has an ordered mesoporous structure with pore diameters ranging from 2.1 to 2.7 nm and a surface area of approximately 1,000 m²/g. The mesoporous silica was suspended in ultrapure water (NACALAI TESQUE, INC., Kyoto, Japan) at a concentration of 0.5 mg/mL using an ultrasonic homogenizer (TAITEC Corporation., Saitama, Japan) operated at 40 W for 5 min and then diluted to the required concentrations with medium. Graphene oxide (Sigma-Aldrich, USA) was also provided by the National Institute of Health Sciences. It was dispersed in water at a concentration of 4 mg/mL and diluted to the required concentrations using medium by pipetting.

### Cell culture

GDL1 cells were established from *gpt* delta mice lung fibroblasts [18] and kindly gifted by Dr Akira Takeiri (Chugai Pharmaceutical., Tokyo, Japan). Cells were passaged every 3–4 days, and cells were dissociated into single cells by treatment with trypsin (Innovative Cell Technologies, San Diego, CA) for 5 min at 37 °C at each passage. The maintenance medium was DMEM (high glucose) (NACALAI TESQUE, INC., Kyoto, Japan) containing 10% fetal bovine serum (Gibco, Carlsbad, CA, USA) and antibiotics (100 units/mL penicillin G and 0.1 mg/mL streptomycin sulfate, Gibco). We also confirmed that the GDL1 cells were able to be recultured after freezing in LaboBanker 2 (Juji Field, Tokyo, Japan) and storage at − 80 °C followed by thawing.

### Evaluation of cell viability of nanomaterials

The effects of mesoporous silica and graphene oxide on the viability of GDL1 cells were evaluated using the trypan blue exclusion method. Briefly, cells (1.0 × 10^6^ cells/well) were incubated for 4–6 hours and then exposed to various concentrations of nanomaterials for 24 h at 37 °C. Cells dissociated with trypsin were treated with 0.4 (w/v) trypan blue solution (Sigma-Aldrich, USA) at a 1:1 ratio and counted using a hemocytometer under a microscope (Olympus Corporation, Tokyo, Japan). All cytotoxicity experiments were performed independently in triplicate.

### Gpt mutation assay

For mutation analysis, GDL1 cells were exposed to appropriate concentrations of nanomaterials for 24 h. After exposure, nanomaterials were removed, and the cells were washed with PBS continuously, trypsinized, and reseeded for subculture. Following reseeding, cells were passaged twice at intervals of 3–4 days. Cells were subsequently harvested and stored at − 80 °C until DNA isolation for mutation assay. High-molecular-weight genomic DNA was extracted from GDL1 cells using a RecoverEase DNA Isolation Kit (Agilent Technologies, Santa Clara, CA, USA), according to the manufacturer’s instructions. Lambda EG10 phages were rescued using the Transpack Packaging Extract (Stratagene, La Jolla, CA, USA). The *gpt* mutation assay was performed according to previously described methods [19]. Briefly, *E. coli* YG6020 was infected with the phage and spread on M9 salt plates containing chloramphenicol (Cm) and 6-thioguanine (6-TG). Plates were incubated for 72 h at 37 °C to select colonies with a plasmid carrying the gene encoding chloramphenicol acetyltransferase, as well as mutated *gpt*. The 6-TG–resistant isolates were cultured overnight at 37 °C in LB broth containing 25 mg/mL of Cm, harvested by centrifugation (7,000 rpm, 10 min), and stored at − 80 °C. Mutational spectra of 6-TG coding sequences were determined using PCR and direct sequencing, and a 739-bp DNA fragment containing *gpt* was amplified by PCR as described previously [19]. Sequence analysis was performed using Eurofins Genomics software (Tokyo, Japan).

### Statistical analysis

For the statistical comparison of the experimental and control groups in the *gpt* mutation assays, data were expressed as means ± standard deviations. The *F*-test was initially performed to evaluate equality of variances. If variances were unequal, Welch’s *t*-test was applied. If not, Student’s *t-*test was used instead. In the case of the mutation spectrum analysis, *P*-values were determined using Fisher’s exact test according to Carr and Gorelick [20]. In any case, *p*-values lower than 0.05 were considered to indicate statistical significance.

## Results

### Cytotoxicity of nanomaterials

**Fig. 1:**
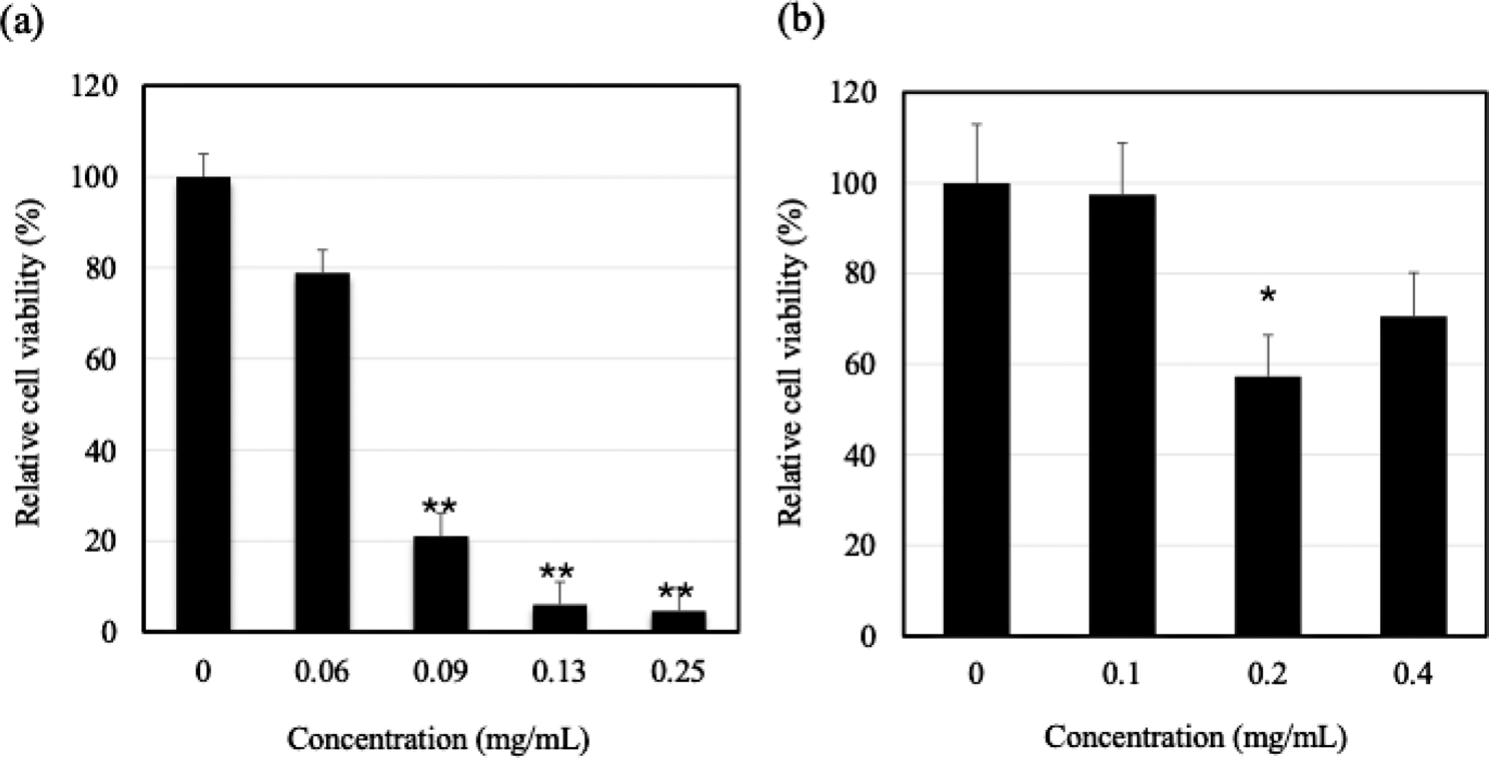
Relative cell viability treatment with mesoporous silica and graphene oxide. Cells were exposed to mesoporous silica and graphene oxide for 24 hours. Cell viability was expressed as a percentage relative to untreated controls. Panel (**a**) shows the results for mesoporous silica, and panel (**b**) shows the results for graphene oxide. Bars indicate mean ± standard deviation (n = 3). *p < 0.05, **p < 0.01 versus control

A 24-hour treatment with mesoporous silica resulted in a concentration-dependent decrease in cell viability, with significant growth inhibition observed at concentrations of 0.09, 0.13, and 0.25 mg/mL compared with the vehicle control (Fig. 1a). Exposure to graphene oxide showed no cytotoxicity at 0.1 mg/mL, with cell viability comparable to that of the control (Fig. 1b). However, cell viability decreased to 57% at 0.2 mg/mL exposure group (*p* < 0.05 vs. control) and 70% in the 0.4 mg/mL exposure group (Fig. 1b). Although a clear concentration-dependent trend was not observed, cytotoxicity was observed in these exposure groups. Based on these results, the concentrations selected for the *gpt* mutation assay were 0.06 and 0.09 mg/mL for mesoporous silica, and 0.2 and 0.4 mg/mL for graphene oxide.

### In vitro mutagenicity of nanomaterials

**Fig. 2:**
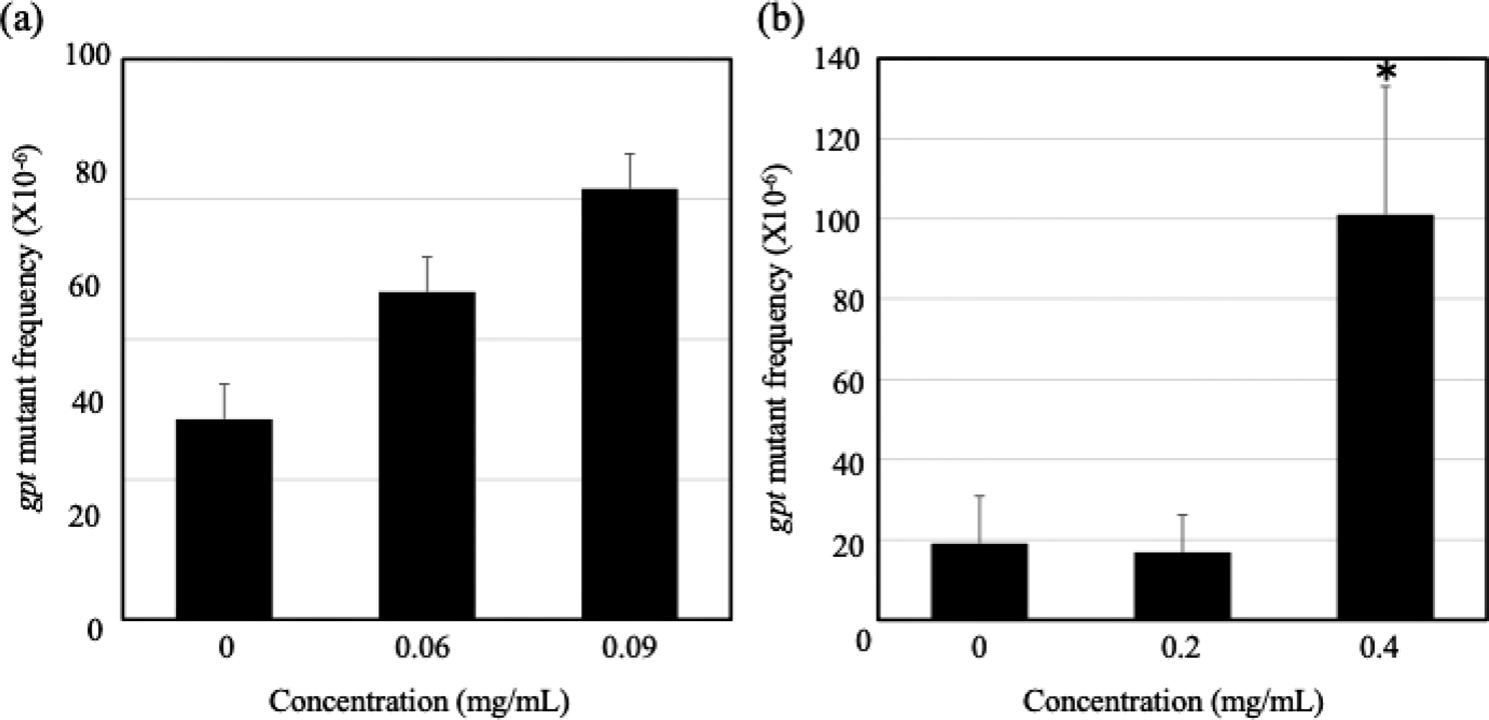
*gpt* mutation frequencies by exposure to mesoporous silica and graphene oxide in GDL1. The data represent the means ± SD. Panel (**a**) shows the results for mesoporous silica, and panel (**b**) shows the results for graphene oxide. * *p <* 0.01 vs. control

The *gpt* mutation frequencies (MFs) in GDL1 cells exposed to mesoporous silica (0.06 and 0.09 mg/mL) increased compared with the control in a dose-dependent manner (Fig. 2a). The MF in the control group was 19.1 × 10⁻^6^, whereas it was 16.9 × 10⁻⁶ and 101 × 10⁻⁶ in the 0.2 and 0.4 mg/mL of graphene oxide exposure groups, respectively, with a statistically significant increase observed at the higher concentration (Fig. 2b).

The classes of *gpt* mutations induced by mesoporous silica are summarized in Table 1. Among the mutation profiles observed in the *gpt* coding sequence in GDL1 cells, the proportion of G: C to A: T transitions significantly increased from 14.3% in the control group to 24.6% in the mesoporous silica exposure group. In addition, exposure induced an increase in G: C to T: A transversions from 0% in the control group to 11.5%. Table 2 summarizes the classes of *gpt* mutations observed in GDL1 cells exposed to graphene oxide. At 0.4 mg/mL, both G: C to C: G transversions and G: C to A: T transitions were significantly elevated, with the frequency of G: C to C: G transversions increasing from 5.6% in the control group to 20.6% in the exposure group, and the proportion of G: C to A: T transitions increasing from 11.1% to 26.5%.

**Table 1:** Classification of *gpt* mutations isolated from GDL1 cells with/without mesoporous silica-treatment

Type of mutation	Control		0.09 mg/mL		*p* value ^a^
	No.of mutants (%)	Specific MF ^b^ (× 10 ^-6^)	No.of mutants (%)	Specific MF ^b^ (× 10 ^-6^)	
G: C to A: T	2 (14.3)	2.20	15 (24.6)	14.4	0.016
A: T to G: C	2 (14.3)	2.20	2 (3.28)	1.92	0.693
G: C to T: A	0 (0.00)	0.00	7 (11.5)	6.70	0.030
G: C to C: G	1 (7.14)	1.10	6 (9.84)	5.74	0.160
A: T to T: A	1 (7.14)	1.10	7 (11.5)	7.66	0.109
A: T toC: G	7 (50.0)	7.70	16 (26.2)	14.4	0.334
Insertion	1 (7.14)	1.10	3 (4.92)	2.87	0.533
Deletion	0 (0.00)	0.00	5 (8.20)	4.79	0.066
Total	14 (100)	15.4	61 (100)	58.4	1.33E-04

a *p* values were determined using Fisher’s exact test according to Carr and Gorelick

b Specific MFs, were calculated by multiplying the total mutation frequency by the ratio of each type of mutation to the total mutation

**Table 2:** Classification of *gpt* mutations isolated from GDL1 cells with/without graphene oxide-treatment

Type of mutation	Control		0.4 mg/mL		*p* value ^a^
	No.of mutants (%)	Specific MF ^b^ (×10^-6^)	No.of mutants (%)	Specific MF b(× 10^-6^)	
G: C to A: T	2 (11.1)	2.15	9 (26.5)	24.3	8.36E-05
A: T to G: C	2 (11.1)	2.15	3 (8.8)	8.16	0.116
G: C to T: A	4 (22.2)	4.31	5 (14.7)	13.6	0.070
G: C to C: G	1 (5.6)	1.08	7 (20.6)	19.1	2.06E-04
A: T to T: A	2 (11.1)	2.15	0 (0.0)	0.00	0.374
A: T to C: G	5 (27.8)	5.39	6 (17.6)	16.3	0.054
Insertion	0 (0.0)	0.00	1 (2.9)	2.72	0.112
Deletion	2 (11.1)	2.15	3 (8.8)	8.16	0.116
Total	18 (100)	1.94	34 (100)	92.5	3.14E-09

a *p* values were determined using Fisher’s exact test according to Carr and Gorelick

b Specific MFs, were calculated by multiplying the total mutation frequency by the ratio of each type of mutation to the total mutation

**Fig. 3:**
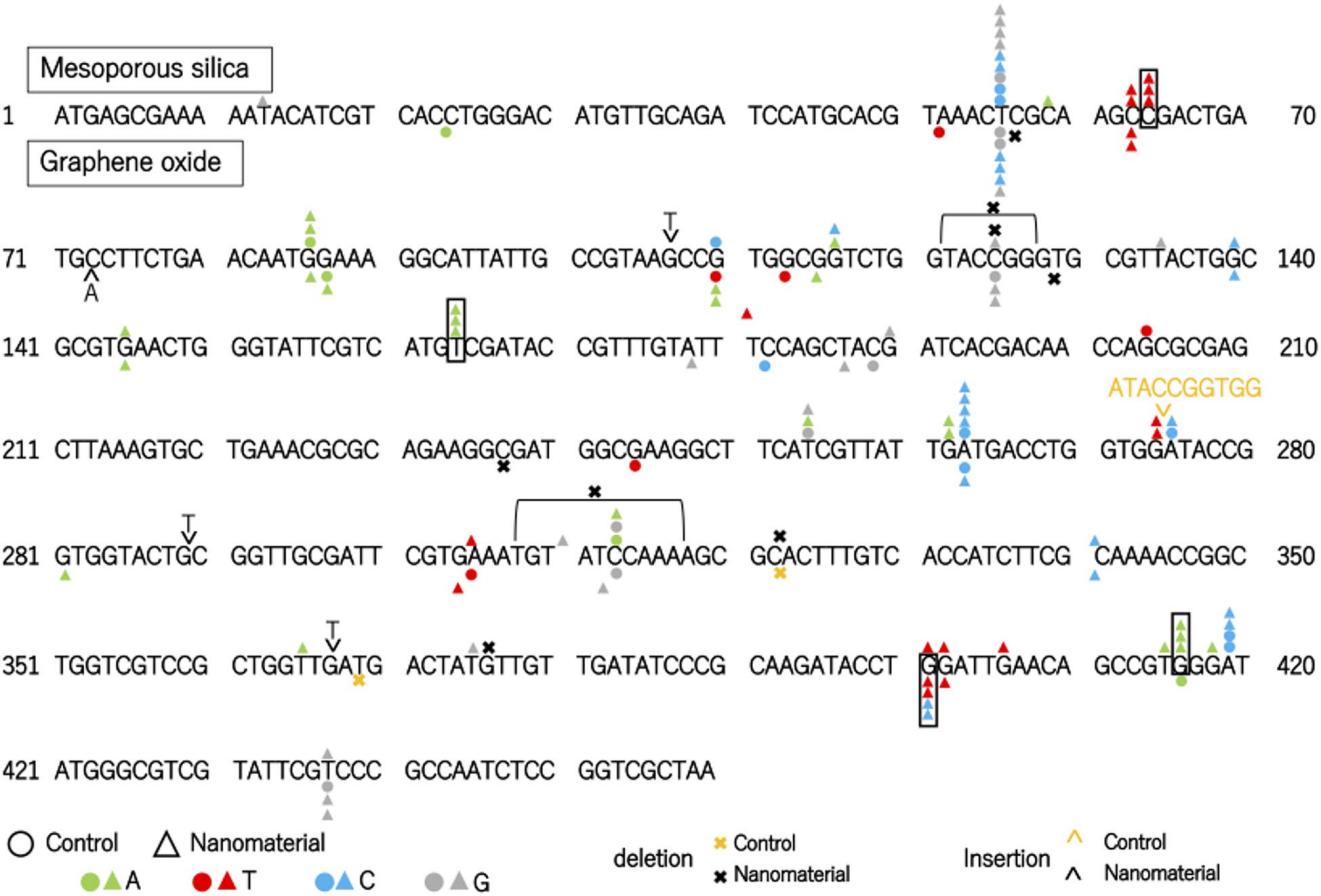
The distribution of spontaneous and nanomaterial-induced mutations in the coding region of gpt in the GDL1. Mutations from mesoporous silica exposure are shown above, and those from graphene oxide exposure are shown below. Circles indicate spontaneous point mutations, and triangles indicate nanomaterial-induced point mutations. Colors represent the substituted nucleobase: adenine (green), thymine (red), cytosine (blue), and guanine (*gray*). Yellow and black symbols indicate spontaneous and nanomaterial-induced deletions/insertions, respectively

The distribution of nanomaterial-induced and spontaneous mutations in the coding region of *gpt* is shown in Fig. 3. Of the 53 mutations induced by mesoporous silica, 10 were G to A transitions and 9 were T to G transversions. Mutation hotspots at positions 64, 164, and 416 were exclusively detected in the mesoporous silica-exposed group, while no such mutations were observed in the control group. Therefore, it is suggested that these mutations can be considered as mesoporous silica induced mutations. Transitions of C to T and transversion of G to C were only observed in the graphene oxide-treated group, therefore it is assumed that these mutations were induced by graphene oxide exposure. Notably, mutations at position 401 were detected exclusively in the graphene oxide group, indicating this site as a potential mutation hotspot.

## Discussion

We investigated the cytotoxicity and genotoxicity of mesoporous silica and graphene oxide using GDL1 cells. Cytotoxicity assays revealed a decrease in cell viability following exposure to both nanomaterials, indicating their cytotoxic potential. The cytotoxicity assay for graphene oxide did not show a clear concentration-dependent effect. This may be attributed to differences in the preparation methods of the exposure solutions: mesoporous silica was dispersed by ultrasonication, while graphene oxide was dispersed by pipetting. The lack of uniform dispersion in the high-dose graphene oxide group may have affected the results.

In the in vitro gene mutation assay test, mesoporous silica significantly increased G: C to T: A and G: C to A: T base substitution mutations. Graphene oxide exposure led to a significant increase in G: C to A: T and G: C to C: G base substitution mutations. One of the known mechanisms through which nanosized particles exert adverse health effects is their ability to generate reactive oxygen species (ROS). Several studies have indicated that the genotoxicity of fine particulate matter, including nanomaterials, is linked to ROS production [21, 22]. For instance, exposure to mesoporous silica has been shown to induce ROS generation and oxidative stress in BEAS-2B cells [23]. In addition, it has been previously reported that exposure of human lung fibroblasts (MRC-5) to silica nanoparticles (SiO₂ NPs) induces ROS production via interactions with NADPH oxidases in the endoplasmic reticulum and mitochondria, disrupting the electron transport chain and generating superoxide anions that subsequently form hydrogen peroxide [24]. Similarly, exposure of human lung fibroblasts (HLF cells) to graphene oxide increases intracellular ROS generation, which can be attenuated by the antioxidant N-acetylcysteine [25]. The mechanism underlying graphene oxide–induced ROS production is suggested to involve carbon radicals on the graphene oxide surface reacting with molecular oxygen to form superoxide anions, potentially triggering oxidative stress [26]. ROS are known to cause oxidative modifications of DNA bases. Among the four DNA bases, guanine is particularly susceptible to oxidation, leading to the formation of 8-oxo-7,8-dihydroguanine (8-oxoguanine). During DNA replication, 8-oxoguanine can mispair with adenine, resulting in G: C to T: A transversions [27]. Therefore, the increase in G: C to T: A transversions caused by mesoporous silica may be attributed to ROS generation.

In general, the G: C to C: G transversion is thought to be a rare event in both spontaneous and chemically induced mutations. However, Kato et al. reported a significant increase in G: C to C: G transversions in *gpt* delta transgenic mice exposed to multi-walled carbon nanotubes (MWCNTs), a type of nanocarbon material similar to graphene oxide [16]. Similar increases in G: C to C: G transversions have also been observed with other nano/microparticles, such as fullerenes (C60), carbon black (CB), and kaolin. In in vitro assay systems, various oxidative stresses caused by sunlight, UV radiation, hydrogen peroxide and peroxyl radicals have frequently been reported to induce G: C to C: G transversions [28, 29]. Graphene oxide has also been shown to induce oxidative stress in A549 cells by generating ROS [30]. While 8-oxo-dG is a common form of DNA damage from oxidative stress, it does not cause G to C transversions because dG is not incorporated opposite 8-oxo-dG [31]. Instead, other oxidative guanine lesion products—such as imidazolone (Iz), oxazolone (Oz), spiroiminodihydantoin (Sp), and guanidinohydantoin (Gh)—have been identified [32, 33]. Among these, Oz, Sp, and Gh are considered key contributors to G to C transversions via translational synthesis mechanisms [32, 33]. Therefore, the G: C to C: G transversions induced by graphene oxide may involve the formation of these oxidized guanine derivatives.

In this study, G:C to A: T transition mutations were also increased following treatment with both nanoparticles. G to A transitions are commonly observed in both spontaneous and chemically induced mutations and are often associated with deamination of 5-methylcytosine or alkylation of guanine [34, 35]. Nitric oxide (NO) causes DNA damage by producing dinitrogen trioxide, which generates diazonium ions that induce DNA deamination and the formation of 8-nitro-dG. This compound can create apurinic sites, preferentially leading to G: C to A: T transition mutations [36, 37]. In a previous study, the lungs of rats exposed to mesoporous silica nanoparticles showed a significant increase in NO levels compared to control rats [38]. Furthermore, graphene oxide has been reported to cause acute lung injury, inflammation, cell apoptosis, and DNA damage through the overproduction of ROS [39]. Graphene oxide exposure also significantly increased ROS production in mouse embryonic fibroblasts [40]. These findings suggest that graphene oxide may trigger inflammatory responses and increase NO production. Therefore, both mesoporous silica and graphene oxide may induce G: C to A: T transition mutations via NO generation.

## Conclusions

We demonstrated that mesoporous silica and graphene oxide exhibit cytotoxic and genotoxic potential in an in vitro assay using GDL1 cells, which were established from *gpt* delta transgenic mice. Based on the prominent mutation spectra observed, it is suggested that oxidative DNA damage and inflammation associated with it may both contribute to their mutagenicity. While the detailed mechanisms underlying the genotoxicity of mesoporous silica and graphene oxide were not investigated in the present study, the findings reported here provide valuable information for elucidating the molecular mechanisms of mutagenicity induced by these nanomaterials and for informing human health risk assessment.

## Data Availability

No datasets were generated or analysed during the current study.
